# Although with intact mucosa at colonoscopy, chagasic megacolons have an overexpression of Gal-3

**DOI:** 10.31744/einstein_journal/2020AO5105

**Published:** 2020-02-27

**Authors:** Mariana Pacífico Garvil, Taíssa Cássia de Souza Furtado, Natália Biagioni de Lima, Maria Vitória Mattar Marteleto, Juliana Barbosa de Faria, Denise Bertulucci Rocha Rodrigues, Sanívia Aparecida de Lima Pereira

**Affiliations:** 1 Universidade de Uberaba UberabaMG Brazil Universidade de Uberaba , Uberaba , MG , Brazil .; 2 Centro de Educação Profissional Universidade Federal do Triângulo Mineiro UberabaMG Brazil Centro de Educação Profissional – Cefores, Universidade Federal do Triângulo Mineiro , Uberaba , MG , Brazil .

**Keywords:** Collagen, Chagas disease, Galectin-3, Mast cells, Megacolon

## Abstract

**Objective:**

To evaluate the density of anti-galectin-3-immunostained cells, collagen percentage, mast cell density and presence of pathological processes in intestinal muscle biopsies of patients.

**Methods:**

Thirty-five patients who underwent intestinal biopsy were selected from 1997 to 2015. Patients were divided into three groups: chagasic patients with mucosal lesion (n=13), chagasic patients with intact mucosa (n=12) and non-chagasic patients with no mucosal lesion (n=10). Histological processing of the biopsied fragments and immunohistochemistry for galectin-3 were performed. Additional sections were stained with hematoxylin and eosin to evaluate the general pathological processes, picrosirius for evaluation of collagen and toluidine blue to evaluate the mast cell density.

**Results:**

Patients of mucosal lesion group had a significantly higher frequency of ganglionitis and myositis when compared to the chagasic patients with intact mucosa and non-chagasic group. The density of anti-galectin-3-immunostained cells was significantly higher in the chagasic patients with intact mucosa group when compared to the non-chagasic group. The group of chagasic patients with intact mucosa presented a higher percentage of collagen in relation to the patients with mucosal lesion and to the non-chagasic group, with a significant difference. There was no significant difference in mast cell density among the three groups.

**Conclusion:**

The higher density of anti-galectin-3-immunostained cells in patients in the chagasic patients with intact mucosa group suggested the need for greater attention in clinical evaluation of these patients, since this protein is associated with neoplastic transformation and progression.

## INTRODUCTION

Chagas disease, described by Carlos Chagas in 1909, is a potentially lethal zoonosis ^( 1 )^ that affects millions of people in Latin America. ^( 2 )^ The World Health Organization (WHO) estimates there are approximately 6 to 7 million people infected worldwide – mostly in Latin America. In Brazil, the number of infected persons is approximately 1,156,821, which is very expressive in the health and social context of the continent, requiring priority and attention on the part of the countries. ^( 3 )^ About 20 to 30% of the infected individuals develop cardiomyopathy and/or digestive syndromes, leading to incapacity or death, with social and economic implications. ^( 4 )^ In the digestive form of Chagas disease, there is destruction of the intramural ganglions and parasympathetic denervation in the entire digestive tract, especially affecting the esophagus and the rectosigmoid. ^( 5 )^

Association between the digestive form of Chagas disease and malignant neoplasms varies from 3.4 to 9.2%. In Chagas disease, neoplasms can arise due to dilation of the organ and the consequent food stasis, triggering prolonged contact between the carcinogen agents and the intestinal mucosa. ^( 6 )^ In this way, the modifications that occur in a chronic infection by *Trypanosoma cruzi (T. cruzi),* especially myoenteric denervation, responsible for the digestive forms of the disease, have a close relation with the etiopathogeny of colorectal carcinogenesis. ^( 7 )^ According to the last global estimate, colon and rectal cancer are the third most common type among men, with 17,380 new cases a year, and the second most common type of cancer in women, with 18,980 new cases in 2018. ^( 8 )^

Various molecules participate in the inflammatory condition in Chagas disease, among which, galactic 3 (Gal-3). ^( 9 , 10 )^ It has been demonstrated that *T. cruzi* uses Gal-3 to interact with laminin, the primary constituent of basal membranes, promoting fixation and entrance of the parasite. ^( 11 )^ On the other hand, the lower expression of Gal-3 favors the multiplication of amastigotes, which suggests that this galectin develops control throughout the chagasic infection. ^( 12 )^ In addition to controlling the multiplication of the amastigotes, it is known that Gal-3 is important in fibrinogenesis, ^( 13 - 15 )^ and is increased in activated myofibroblasts and monocytes, besides being important for the activation of mast cells. ^( 16 )^ Mast cells release tryptase and thrombin, which increase the differentiation of human fibrocytes, leading to the formation of collagen fibers in damaged tissues. ^( 17 )^

Although Gal-3 seems to be important for the control of *T. cruzi* , ^( 12 )^ this galectin has been associated with the malignization of a few lesions, having been suggested that the evaluation of this galectin could work as a marker for tumor lesions. ^( 18 )^ Nevertheless, the role of Gal-3 in neoplasms is not yet well understood. Generally, it is related to the cell-cell adhesion and cell-matrix adhesion, cellular polarity, motility, activation, differentiation, transformation, signaling, regulation of the adaptive/innate immunity, and angiogenesis. ^( 19 )^

In colorectal cancers, it has been shown that the large expression of Gal-3 promotes the beginning and progression of the tumors, and is associated with metastasis and with a poor prognosis. ^( 20 )^ Patients with increased expression of Gal-3 die more frequently or have a greater tendency towards relapses. Despite this, the risk of death is reduced in patients with the absence or low expression of Gal-3. ^( 21 )^

In the present study, we raised the hypothesis that the patients with chagasic megacolon show a greater frequency of pathological processes, such as myositis, and ganglionitis; a greater percentage of collagen; and lower density of mast cells in the muscular layer of the colon. Additionally, we believe that the Chagas disease patients present with greater expression of Gal-3, especially in areas with a lesion visible on colonoscopy. The large expression of Gal-3 in these lesions would indicate a greater possibility of malignant transformation and metastasis of cancerous lesions. Thus, the evaluation of Gal-3 could contribute as an additional factor in determining the malignancy potential of the lesions found in chagasic megacolons.

## OBJECTIVE

To evaluate the frequency of pathological processes, the percentage of collagen, the density of mast cells, and the density of cells immunostained by anti-Gal-3 in the intestinal muscles of patients biopsied with chagasic megacolon, who presented with an intact or damaged mucosa on colonoscopy.

## METHODS

### Patient selection

Thirty-five colon fragments were selected from the biopsies of 35 patients aged between 44 and 85 years. Patients were divided into three groups, according to the description present in the reports: Chagas disease patients with mucosal lesion (CML) (n=13), Chagas disease patients with intact mucosa (CIM) (n=12), and non-chagasic patients with no mucosal lesions (NC) (n=10). Individuals were homogenized as per age and sex.

The inclusion criteria were: (1) for the CML Group: Chagas disease patients, presenting with megacolon with an ulcerated intestinal mucosa and/or mucosal hypotrophy; (2) for the CIM Group: chagasic patients, presenting with megacolon and intact intestinal mucosa; (3) for the NC Group: non-chagasic patients with intestinal infarction, who did not present with any inflammatory lesion of the intestinal mucosa or muscles. For this group, the fragments were removed from the region close to the infarction.

Excluded from the study were patients with megacolon from other etiologies, aged under 18 years, Chagas disease patients with no megacolon, and intestinal mucosa lesions with no chagasic megacolon.

The present project was approved by the Research Ethics Committee of the *Universidade Federal do Triângulo Mineiro* (UFTM), with CAAE: 22725013.4.0000.5154 and opinion no. 735.931. After approval, a longitudinal retrospective study was carried out with an analysis of protocols of complete biopsies performed with clinical indication in a private laboratory in the city of Uberaba (MG, Brazil), between 1997 and 2015.

### Histological processing

The biopsied fragments of the colon muscularis propria were included in paraffin and sliced in a microtome, obtaining 5µm-thick slices. These slices were placed on glass slides and stained with the following elements: hematoxylin and eosin, for evaluation of general pathological processes; picrosirius and toluidine blue, for evaluation of collagen and mast cells, respectively. The other slice was placed on a silanized slide for immunohistochemical processing for Gal-3.

### Processing and immunohistochemical evaluation for galectin-3

For the immunohistochemical processing of Gal-3, the slices were deparaffinized, rehydrated, and washed with ultrapure water for 5 minutes at room temperature. Then, the antigenic retrieval with humid heat was done, with 0.01 M citric acid and pH 6 for 30 minutes. Next, the slices were incubated with 2% phosphate buffered saline/bovine serum albumin (PBS/BSA) during 30 minutes, to block unspecific bonds.

The anti-Gal-3 antibodies were diluted in 2% PBS/BSA at a concentration of 1:75 (R&D, Minnesota, USA). Further, the slices were incubated for 18 hours (overnight) at 4°C with diluted primary antibody, and posteriorly were washed twice with PBS and Tween 20 at 0.05%. The slices were the treated with methanol and hydrogen peroxide (H _2_ O _2_ ) at 3% for 15 minutes, to block the endogenous peroxidase of the tissues. For detection of the antibody, the technique used was avidin-biotin conjugated with peroxidase (ABC) utilizing the lsab-plus (DAKO, Carpinteria, USA) kit. The complex was incubated for 30 minutes at room temperature of the laboratory (22°C - 25°C), and washed with PBS in the same manner as before.

The slides were developed with diaminobenzidine (DAB) (0.5mg/mL) and H _2_ O _2_ 0.05% at room temperature, protected from light. Soon afterwards, the slices were washed with distilled water, counterstained with Harris hematoxylin, and mounted with Entellan.

The slides on which the immunohistochemical technique was performed for Gal-3, were analyzed under common light microscopy and a 63x lens, utilizing the Eclipse (Nikon, Berlin, Germany) microscope. Counting of cells immunostained with Gal-3 was carried out in all the fields of intestinal muscles. With the help of a micrometer blade, the area of the field on the 63x lens was calculated. Next, this area (0.14µm ^2^ ) was multiplied by the total number of fields analyzed to obtain the total area analyzed. With the total number of immunostained cells and the total area analyzed, the density of immunostained cells was calculated and was expressed in number of cells per µm ^2^ .

### Determination of the collagen percentage

To evaluate the determination of the percentage of collagen in the intestinal muscles, slides stained by picrosirius were used. Picrosirius allows visualization of collagens type I and type III, enabling the qualitative analysis of the collagen fibers of the connective tissue, by means of different color interference, intensity, and birefringence of the stained tissues, distinguishing primarily type I and type III fibers. Type I fibers are presented thick, highly bifrengent and red, while type III fibers are in thin bundles, with weak birefringence and yellow-greenish. Analysis of the collagen was performed in all the fields where it was possible to observe muscles. Images were captured using a common light microscope, Axio 4.1 (ZEISS, Berlin, Germany), an Axiocam (ZEISS, Berlin, Germany) image-capturing camera, a computer, and the Axiovision 4.8 (ZEISS, Berlin, Germany) software. The images seen on the microscope were transmitted to the computer screen. For this analysis, the 40x lens and a polarizing filter were used. In the polarized image, collagen showed birefringence with yellowish, reddish, or greenish coloring, and is marked by the examiner with the help of a cursor. In this way, the software automatically determined the percentage of collagen per field.

### Determining mast cell density

To determine the mast cell density, slides stained by toluidine blue were used. The slices were deparaffinized, washed in distilled water, stained with fuchsin-orange G, and quickly immersed in 60% alcohol. Next, the slides were rapidly immersed in toluidine blue and washed in running water. The images were analyzed on a common light microscope (Nikon, Berlin, Germany), with a 40x lens.

Mast cell count was carried out in all fields of the slide. With the help of a micrometer slide, the area of each field was calculated, and this area was multiplied by the total number of fields analyzed, in order to obtain the total area analyzed. With the total number of mast cells and the total area analyzed, the density of mast cells was calculated, expressed in number of mast cells per µm ^2^ .

### Assessment of the pathological processes

The evaluation of the general pathological processes was done with the slides stained by hematoxylin and eosin. The following pathological processes were assessed as being present or absent: ganglionitis, myositis, congestion, hemorrhage, degeneration, and necrosis, with a score of zero and one, where zero corresponded to absent, and one to present.

The histochemical and immunohistochemical analyses were conducted by a single blinded examiner.

### Statistical analysis

The statistical analysis was done by means of the GraphPad Prism 5 (GraphPad, San Diego, California, USA) software. Kolmogorov-Smirnov test was used to evaluate normality. In cases with normal distribution, for comparison among the three groups, the variance analysis test (ANOVA) was used, and in cases of non-normal distribution, Kruskal-Wallis test was employed. For comparison among chagasic (CIM+CML) and non-chagasic (NC) patients with normal distribution, Student’s *t* test was used, and for non-normal distribution, the Mann-Whitney test. To compare between sexes, the χ ^2^ test was employed. For correlations, Spearman’s correlation test was used. The adopted level of significance was 5% (α<0.05).

## RESULTS

When evaluating individuals from the three groups, there was no significant difference as to age and sex, demonstrating a homogeneous distribution among the groups ( Table 1 ).

**Table 1:** Distribution of age and sex of the individuals in the groups chagasic patients with mucosal lesions, chagasic patients with intact mucosa, and non-chagasic individuals

	CML	CIM	NC
	(n=13)	(n=12)	(n=10)
Age*	67 (85-44)	66 (82-54)	69 (80-56)
Sex (male: female) ^#^	4:9	4:8	6:4

^*^ Variance analysis test; F=0.8458; p>0.050; ^#^ test χ ^2^ =2,350.2; p=0.309.

CML: Chagasic patient with mucosal lesion; CIM: Chagasic patient with intact mucosa; NC: Non-chagasic individuals with no mucosal lesion.

**Figure 1:**
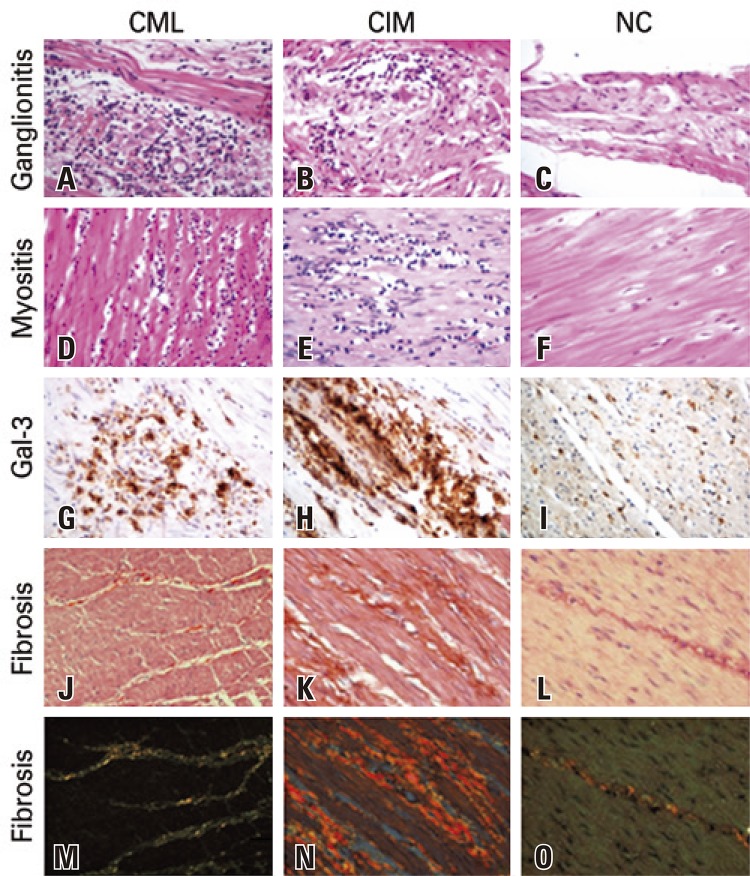
Ganglionitis, myositis, Gal-3 expression, and fibrosis in the muscularis propria of the colon of patients in the groups of chagasic patients with mucosal lesions, chagasic patients with intact mucosa, and non-chagasic. A) Ganglionitis in the group of chagasic patients with mucosal lesion (CML); B) Ganglionitis in the group of chagasic patients with intact mucosa (CIM); C) Absence of ganglionitis in the group of chagasic patients non-chagasic; D) Myositis in the group of chagasic patients with mucosal lesions (hematoxylin and eosin, 400x); E) Myositis in the group of chagasic patients with intact mucosa (hematoxylin and eosin, 400x); F) Absence of myositis in the group of chagasic patients non-chagasic (hematoxyllin and eosin, 400x); G) Expression of Gal-3 in the group of chagasic patients with mucosal lesions (immunohistochemistry, 400x); H) Expression of ganglionitis 3 in the group of chagasic patients with mucosal lesions (immunohistochemistry, 400x); I) Expression of ganglionitis in the group non-chagasic (immunohistochemistry, 400x); J) Fibrosis in the group of chagasic patients with mucosal lesion (picrosirius, 1600x); K) Fibrosis in the group of chagasic patients with intact mucosa (picrosirius, 1600x); L) Fibrosis in the group non-chagasic (picrosirius, 1600x); M) Fibrosis in the group of chagasic patients with mucosal lesion (polarized picrosirius image, 1600x); N) Fibrosis in the group of chagasic patients with intact mucosa (picrosirius, polarized image, 1600x); O) Discreet fibrosis in the group non-chagasic (picrosirius, polarized image, 1600x)

The CML Group presented with a significantly higher frequency of myositis (p=0.0250) and of ganglionitis (p=0.0390) when compared to the others. The chagasic individuals (CML + CIM) presented with a significantly higher frequency of myositis (p=0.0216) and of ganglionitis (p=0.0140) when compared to those of the NC Group ( Figure 1 and Table 2 ).

**Table 2:** Distribution of the general pathological processes in the groups of chagasic patients with mucosal lesions, chagasic patients with intact mucosa, and non-chagasic individuals

Pathological process	CML	CIM	NC
	n=13 (100%)	n=12 (100%)	n=10 (100%)
Congestion ^≠^	6 (46.15)	10 (83.33)	6 (60)
Hemorrhage ^#^	0 (0)	2 (16.67)	1 (10)
Degeneration ^†^	3 (23.08)	4 (33.33)	2 (20)
Necrosis ^‡^	0 (0)	0 (0)	1 (10)
Myositis* ^£^	10 (76.92)	6 (50)	2 (20)
Ganglionitis* ^§^	6 (46.15)	5 (41.67)	0 (0)

* indicates statistical difference; ^≠^ χ ^2^ test=3,744.2, p=0.153; ^#^ Fisher’s exact test (CML + CIM *versus* NC); p=1; ^†^ χ ^2^ test=0.582; p=0.747; ^‡^ Fisher’s exact test (CML + CIM *versus* NC), p=1; ^£^ χ ^2^ test=7,347.2, p=0.025; ^§^ χ ^2^ test=6,475.2, p=0.039.

CML: chagasic patients with mucosal lesion; CIM: chagasic patients with intact mucosa; NC: non-chagasic individuals with no mucosal lesion.

**Figure 2:**
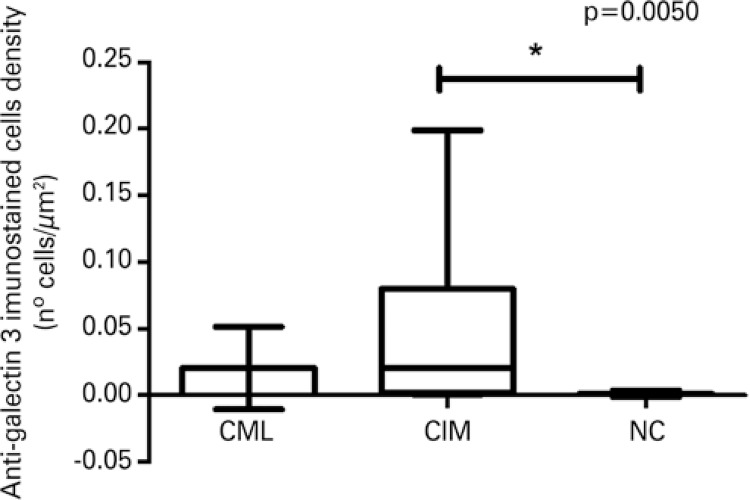
Density of cells immunostained with anti-galectin-3 among the groups of chagasic patients with mucosal lesions, chagasic patients with intact mucosa, and non-chagasic individuals * Statistical difference according to the Kruskal-Wallis test, K=10.58; p=0.0050. CML: chagasic patients with mucosal lesion; CIM: chagasic patients with intact mucosa; NC: non-chagasic individuals with no mucosal lesions.

The density of immunostained cells by anti-Gal-3 was significantly greater in the chagasic patients (CML + CIM) when compared to the NC Group (p=0.0032; data not shown) and significantly greater in the CIM Group when compared to the NC Group (p=0.0050) ( Figures 1 and 2 ).

**Figure 3:**
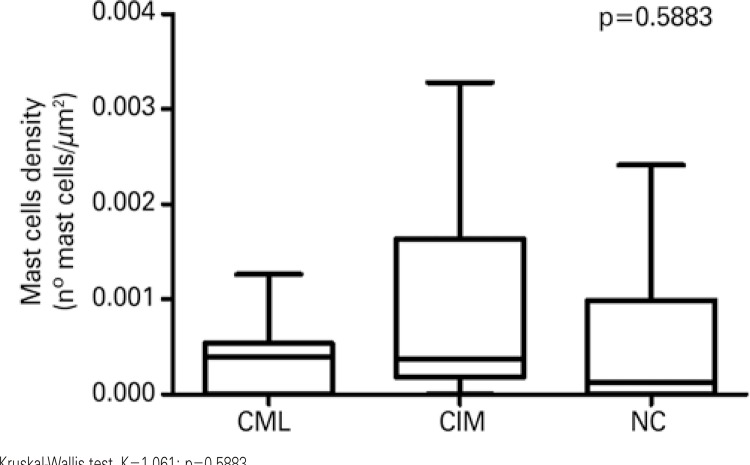
Density of mast cells among the groups of chagasic patients with mucosal lesions, chagasic patients with intact mucosa, and non-chagasic individuals Kruskal-Wallis test, K=1.061; p=0.5883. CML: chagasic patients with mucosal lesion; CIM: chagasic patients with intact mucosa; NC: non-chagasic individuals with no mucosal lesions.

As to the density of mast cells, there was no significant difference among the three groups (p=0.5883) ( Figure 3 ).

**Figure 4:**
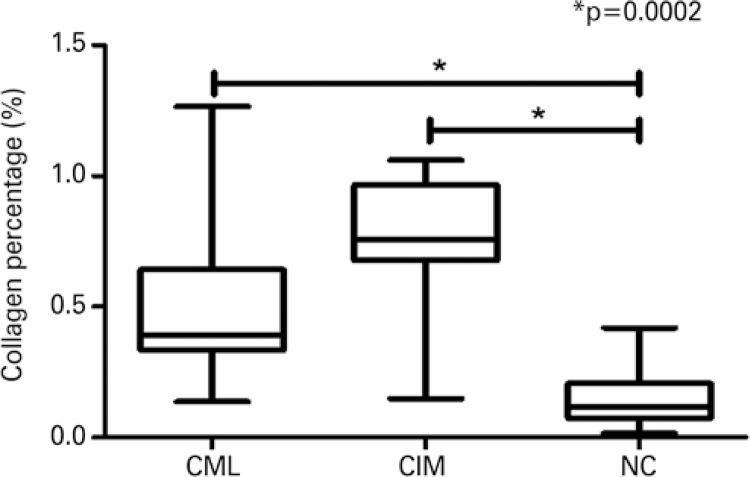
Percentage of collagen among the groups of chagasic patients with mucosal lesions, chagasic patients with intact mucosa, and non-chagasic individuals * Statistical difference according to the Kruskal-Wallis test, K=17.09; p=0.0002. CML: chagasic patients with mucosal lesion; CIM: chagasic patients with intact mucosa; NC: non-chagasic individuals with no mucosal lesions.

The chagasics patients (CML + CIM) presented percentage of collagen significantly greater than the patients in the NC Group (p=0.0014; data not shown). The percentage of collagen was significantly greater in the CIM and CML Groups when compared to the NC Group (p=0.0002) with a predominance of type I collagen, typical of a chronic inflammatory process, seen in reddish color under polarized light ( Figures 1 and 4 ).

Regardless of the integrity of the mucosa, chagasic patients presented with a greater density of immunostained cells by anti-Gal-3, and a greater percentage of collagen when compared to non-chagasic individuals.

## DISCUSSION

Although the biological role of Gal-3 is not yet totally known, we do know that it is associated with adhesion, cell proliferation, and apoptosis. Additionally, it participates in pathological processes, such as cell hypertrophy, inflammation, and neoplastic transformation. ^( 22 )^ In Chagas disease, Gal-3 increases the adherence of *T. cruzi* to the extracellular matrix, allowing the parasites to accumulate in these locations before invading host cells. ^( 23 )^

Gal-3 also presents with pro-inflammatory action, even when participating in the fixation of *T. cruzi* to tissues, and is increased in the hearts of mice chronically infected by the parasite. ^( 12 )^ As in the present study, chagasic patients presented with a significantly greater density of cells immunostained by anti-Gal-3 when compared to non-chagasic individuals. We suggest that this increase is a consequence of the inflammatory condition caused by the parasite. This finding corroborates a previous study carried out by our team, which demonstrated a greater density of Gal-3 in the megacolon of chagasic patients when compared to non-chagasic individuals, but without taking into consideration the presence of mucosal lesions. ^( 24 )^ Since the CIM Group patients showed a significantly greater Gal-3 density when compared to the NC Group, we reinforced the hypothesis that this galectin would be increased due to the chagasic infection, and not because of possible mucosal lesions, which could affect the muscularis propria of the colon with resulting inflammation.

The most frequent modifications in the chagasic megacolon are myositis, ganglionitis, periganglionitis, neuritis, perineuritis, denervation, and degenerative phenomena of the neurons, ^( 25 )^ which corroborates the findings of the present study, since chagasic patients (CML + CIM) have a significantly greater presence of ganglionitis and myositis, when compared to the patients from the NC Group. When the comparison was made among the three groups, the CML Group had a significantly greater presence of ganglionitis and myositis, when compared to the other Groups. In this way, the inflammatory process in the ganglia and in the intestinal musculature of the CML Group should also occur by the propagation of the mucosal inflammatory process, and not only by the infection with *T. cruzi* in the intestinal muscles. Additionally, since the chagasic patients presented with a greater density of cells immunostained for Gal-3, we believe that this galectin presents pro-inflammatory action and therefore could be contributing towards the condition of myositis and ganglionitis. The present study was the first to compare the frequency of myositis and of ganglionitis among chagasic patients with mucosal lesions and intact mucosa seen in colonoscopy.

Gal-3 is also associated with fibrosis, participating in the activation of myofibroblasts. It has already been shown that the fibrotic activity of the tissue growth factor (TGF-β) only occurs in the presence of Gal-3, ^( 26 )^ and the absence of Gal-3 is related to the interruption of the fibrogenic process. ^( 14 )^ Additionally, this galectin provokes an increase in number and degranulation of mast cells. ^( 16 )^ Mast cells release tryptase and thrombin, which increase the differentiation of human fibrocytes that, in turn, lead to the formation of collagen fibers in damaged tissues. ^( 17 )^ In the present study, chagasic patients (CML+CIM), who also presented with a higher density of Gal-3, showed a percentage of collagen significantly greater than the NC Group, which corroborates the literature, since it has already been demonstrated in mice that *T. cruzi* can be the factor direct or indirectly responsible for molecular modification in different tissues and organs, enabling the induction of cell lesions, inflammatory response, and fibrosis mediated by Gal-3. ^( 27 )^ Nonetheless, our study was the first to associate Gal-3 with fibrosis in Chagas disease in humans.

When individually analyzing the groups, the percentage of collagen was significantly greater in the CIM and CML Groups, when compared to the NC Group, with a predominance of collagen type I, typical of a chronic inflammatory process, seen as redish on polarized light. Although we found no studies evaluating the percentage of collagen comparing areas with or without intestinal mucosa lesion, we know that in Chagas disease, there is a greater percentage of collagen in the affected organs. ^( 28 )^ Additionally, during inflammation, there is release of substances that present with lytic action, such as metaloproteinases-2, proteolytic enzymes that are involved in the lysis of collagen, ^( 29 )^ which perhaps justifies the fact that CML Group presented with a relatively smaller percentage of collagen than CIM Group, probably as a result of the inflammatory response in these sites.

It is known that during the chronic phase of Chagas disease, there is an increase in the number of mast cells in various locations. ^( 16 )^ Studies carried out in bone marrow of mice demonstrated that Gal-3 is related to increased number and degranulation ^( 16 )^ of these cells. Therefore, we raised the hypothesis that chagasic patients would present with a higher density of mast cells, since they already presented with a higher density of cells immunostained by Gal-3. Nevertheless, in the present study, there was no statistical difference relative to the density of mast cells among the groups evaluated. In this way, a large part of the mast cells would have undergone degranulation, impeding their identification.

In addition to being involved in the inflammatory process in Chagas disease, promoting the fixation of the parasite in the tissues, degranulation of mast cells, and fibrosis, Gal-3 is also associated with neoplasms in several sites, since it is associated with increased invasive capacity, decrease in apoptosis, angiogenesis, and tumor growth. ^( 30 )^ In colorectal cancers, the large expression of Gal-3 promotes the beginning and progression of the tumors, and is associated with metastasis and with a poor prognosis. ^( 20 )^ Also, patients with increased expression of Gal-3 die with greater frequency or have a greater tendency to present with relapses. On the other hand, the risk of death is reduced in patients with a low expression or absence of Gal-3. ^( 21 )^ Therefore, as we found a greater density of Gal-3 in CIM group when compared to NC Group, we suggested that chagasic patients, even without presenting clinical lesion on colonoscopy, should be periodically accompanied in order to prevent the development of colon cancer, as they showed more Gal-3 in these locations.

## CONCLUSION

Chagasic patients with intact mucosa on colonoscopy presented with overexpression of Gal-3 and a greater percentage of collagen in the colon muscles, when compared to chagasic patients with mucosal lesions, and to non-chagasic individuals. The greater density of cells immunostained by anti-galectin-3 in patients with intact mucosa suggests the need for clinical follow-up of these patients, since this protein is associated with neoplastic transformation and progression. Nevertheless, new studies should be done to understand the mechanism of action of Gal-3 in colorectal cancers associated with Chagas disease, in order to suggest indications and frequency of colonoscopies in these patients.
