# Supplementary material for: Although with intact mucosa at colonoscopy, chagasic megacolons have an overexpression of Gal-3
Source: Einstein (Sao Paulo). 2020 Feb 27;18:eAO5105. doi: 10.31744/einstein_journal/2020AO5105 (PMC7046340; doi:10.31744/einstein_journal/2020AO5105)
Supplement: Translation

# Translation: Embora com mucosa íntegra à colonoscopia, megacólons chagásicos apresentam superexpressão de Gal-3

**Authors:** Mariana Pacífico Garvil, Taíssa Cássia de Souza Furtado, Natália Biagioni de Lima, Maria Vitória Mattar Marteleto, Juliana Barbosa de Faria, Denise Bertulucci Rocha Rodrigues, Sanívia Aparecida de Lima Pereira

**Affiliations:** 1 Brasil Universidade de Uberaba, Uberaba, MG, Brasil .; 2 Brasil Centro de Educação Profissional – Cefores, Universidade Federal do Triângulo Mineiro, Uberaba, MG, Brasil .

**Keywords:** Colágeno, Doença de Chagas, Galectina-3, Mastócitos, Megacólon

## Abstract

**Objetivo:**

Avaliar a densidade de células imunomarcadas por anti-galectina-3, a percentagem de colágeno, a densidade de mastócitos e a presença de processos patológicos na musculatura intestinal de pacientes biopsiados.

**Métodos:**

Foram selecionados 35 pacientes submetidos à biópsia de intestino entre 1997 a 2015. Os pacientes foram divididos em três grupos: chagásicos com lesão de mucosa (n=13), chagásicos com mucosa íntegra (n=12) e não chagásicos sem lesão de mucosa (n=10). Foram realizados processamento histológico dos fragmentos biopsiados e imunohistoquímica para galectina-3. Cortes adicionais foram corados por hematoxilina e eosina, para avaliar os processos patológicos gerais, pelo picrosírius, para avaliação do colágeno, e pelo azul de toluidina, para avaliar a densidade de mastócitos.

**Resultados:**

Os pacientes do grupo chagásicos com lesão de mucosa apresentaram frequência significativamente maior de ganglionite e miosite quando comparados aos dos grupos chagásico com mucosa íntegra e não chagásicos. A densidade das células imunomarcadas por anti-galectina-3 foi significativamente maior no grupo chagásicos com mucosa íntegra quando comparada ao grupo não chagásico. O grupo de chagásicos com mucosa íntegra apresentou maior percentagem de colágeno em relação aos grupos chagásicos com mucosa lesada e ao grupo de não chagásicos, com diferença significativa. Não houve diferença significativa com relação à densidade de mastócitos entre os três grupos.

**Conclusão:**

A maior densidade de células imunomarcadas por anti-galectina-3 nos pacientes do grupo chagásico com mucosa íntegra sugere a necessidade de maior atenção na avaliação clínica desses pacientes, uma vez que essa proteína está associada com transformação e progressão neoplásica.

## INTRODUÇÃO

A doença de Chagas, descrita por Carlos Chagas, em 1909, é uma zoonose potencialmente letal,^( 1 )^que afeta milhões de pessoas na América Latina.^( 2 )^A Organização Mundial da Saúde (OMS) estima em aproximadamente 6 a 7 milhões o número de pessoas infectadas em todo o mundo − a maioria na América Latina. No Brasil, o número de pessoas infectadas é em torno de 1.156.821, o qual é muito expressivo no contexto sanitário e social do continente, requerendo prioridade e atenção por parte dos países.^( 3 )^Cerca de 20 a 30% dos indivíduos infectados desenvolvem cardiomiopatia e/ou síndromes digestivas, levando à incapacidade ou à morte, com implicações de ordem social e econômica.^( 4 )^Na forma digestiva da doença de Chagas, existe destruição de gânglios intramurais e desnervação parassimpática em todo o trato digestório, afetando especialmente o esôfago e o retossigmoide.^( 5 )^

A associação entre a forma digestiva da doença de Chagas e neoplasias malignas varia de 3,4 a 9,2%. Na doença de Chagas, as neoplasias podem surgir devido à dilatação do órgão e à consequente estase alimentar, desencadeando contato prolongado entre os agentes cancerígenos e a mucosa intestinal.^( 6 )^Dessa maneira, as alterações que ocorrem na infecção crônica por *Trypanossoma cruzi (T. cruzi),* em especial a denervação mioentérica, responsável pelas formas digestivas da doença, guardam estreita relação com a etiopatogenia da carcinogênese colorretal.^( 7 )^De acordo com a última estimativa mundial, o câncer de cólon e reto configura-se como o terceiro tipo mais comum entre os homens, com 17.380 casos novos, e o segundo tipo de câncer mais comum nas mulheres, sendo 18.980 casos novos para o ano de 2018.^( 8 )^

Várias moléculas participam do quadro inflamatório na doença de Chagas, dentre elas a galectina-3 (Gal-3).^( 9 , 10 )^Já foi demonstrado que *T. cruzi* utiliza a Gal-3 para interagir com a laminina, principal constituinte das membranas basais, promovendo a fixação e a entrada do parasita.^( 11 )^Por outro lado, a menor expressão de Gal-3 favorece a multiplicação dos amastigotas, o que sugere que essa galectina desempenhe controle no decorrer da infecção chagásica.^( 12 )^Além de controlar multiplicação dos amastigostas, sabe-se que a Gal-3 é importante na fibrogênese,^( 13 - 15 )^encontrando-se aumentada em miofibroblastos e monócitos ativados, além de ser importante para ativação de mastócitos.^( 16 )^Os mastócitos liberam triptase e trombina, que aumentam a diferenciação de fibrócitos humanos, levando à formação de fibras colágenas em tecidos danificados.^( 17 )^

Embora a Gal-3 pareça ser importante para o controle de *T. cruzi* ,^( 12 )^essa galectina foi associada com a malignização de algumas lesões, sendo sugerido, inclusive, que a avaliação dessa galectina poderia funcionar como marcador para lesões tumorais.^( 18 )^No entanto, o papel da Gal-3 nas neoplasias ainda não é bem compreendido. Geralmente, está relacionada com adesão célula-célula e adesão célula-matriz, polaridade celular, motilidade, ativação, diferenciação, transformação, sinalização, regulação da imunidade adaptativa/inata e angiogênese.^( 19 )^

Em cânceres colorretais, já foi demonstrado que a grande expressão de Gal-3 promove o início e a progressão dos tumores, estando associada com metástase e com mau prognóstico.^( 20 )^Pacientes com aumento na expressão de Gal-3 vão a óbito com mais frequência ou possuem maior tendência de recidivas. No entanto o risco de morte é reduzido em pacientes com ausência ou baixa expressão de Gal-3.^( 21 )^

No presente estudo, levantamos a hipótese de que os pacientes com megacólon chagásico apresentam maior frequência de processos patológicos, como miosite e ganglionite; maior percentagem de colágeno; e maior densidade de mastócitos na camada muscular do cólon. Além disso, acreditamos que os pacientes chagásicos apresentam maior expressão de Gal-3, principalmente em áreas com lesão de mucosa visível à colonoscopia. A grande expressão de Gal-3 nessas lesões indicaria maior probabilidade de transformação maligna e de metástase de lesões cancerígenas. Assim, a avaliação de Gal-3 poderia contribuir como um fator adicional para determinar o potencial de malignidade das lesões encontradas nos megacólons chagásicos.

## OBJETIVO

Avaliar a frequência de processos patológicos, a percentagem de colágeno, a densidade de mastócitos e a densidade de células imunomarcadas por anti-Gal-3 na musculatura intestinal de pacientes biopsiados com megacólon chagásico que apresentavam mucosa íntegra ou lesada à colonoscopia.

## MÉTODOS

### Seleção dos pacientes

Foram selecionados 35 fragmentos de cólon provenientes de biópsias de 35 pacientes com idade entre 44 a 85 anos. Os pacientes foram divididos em três grupos, de acordo com a descrição presente nos laudos: chagásicos com lesão de mucosa (CLM) (n=13), chagásicos com mucosa íntegra (CMI) (n=12) e não chagásicos sem lesões de mucosa (NC) (n=10). Os indivíduos foram homogeneizados de acordo com idade e sexo.

Os critérios de inclusão foram: (1) para o Grupo CLM: pacientes chagásicos, apresentando megacólon com a mucosa intestinal ulcerada e/ou com hipotrofia de mucosa; (2) para o Grupo CMI: pacientes chagásicos, apresentando megacólon com mucosa intestinal íntegra; (3) para o Grupo NC: pacientes com infarto intestinal, não chagásicos e que não apresentavam qualquer lesão inflamatória de mucosa ou na musculatura intestinal. Para este grupo, os fragmentos foram retirados na região próxima ao infarto.

Foram excluídos do estudo os pacientes com megacólon de outras etiologias; pacientes com idade inferior a 18 anos; chagásicos sem megacólon e pacientes que apresentavam lesões de mucosa intestinal sem apresentar megacólon chagásico.

O presente projeto foi aprovado pelo Comitê de Ética em Pesquisa da Universidade Federal do Triângulo Mineiro (UFTM), sob o número CAAE: 22725013.4.0000.5154 e parecer 735.931. Após aprovação, foi realizado estudo retrospectivo longitudinal com a análise de protocolos de biópsias completas realizadas com indicação clínica em um laboratório particular da cidade de Uberaba (MG), no período entre 1997 a 2015.

### Processamento histológico

Os fragmentos biopsiados da camada muscular própria do colón foram incluídos em parafina e cortados em micrótomo, obtendo-se cortes de 5µm de espessura. Esses cortes foram colocados sobre lâminas de vidro e corados pelas seguintes colorações: hematoxilina e eosina, para avaliação de processos patológicos gerais; picrosírius, para avaliação do colágeno; azul de toluidina, para avaliação dos mastócitos. O outro corte foi colocado em lâmina silanizada para o processamento imunohistoquímico para Gal-3.

### Processamento e avaliação imunohistoquímica para Galectina-3

Para o processamento da imunohistoquímica para Gal-3, os cortes foram desparafinados, reidratados e lavados com água ultrapura por 5 minutos à temperatura ambiente. Posteriormente, foi realizada a recuperação antigênica ao calor úmido, com ácido cítrico 0,01M e pH 6, durante 30 minutos. Em seguida, os cortes foram incubados com tampão fosfato-salino/albumina de soro bovino (PBS/BSA) 2%, durante 30 minutos, para bloqueio das ligações inespecíficas.

Os anticorpos anti-Gal-3 foram diluídos em PBS/BSA 2% na concentração de 1:75 (R&D, Minnesota, EUA). Em seguida, os cortes foram incubados por 18 horas a 4°C com anticorpo primário diluído, sendo posteriormente lavados duas vezes com PBS e *Tween* 20 a 0,05%. Os cortes foram, então, tratados com metanol e peróxido de hidrogênio (H_2_O_2_) a 3% durante 15 minutos para bloqueio da peroxidase endógena dos tecidos. Para a detecção do anticorpo, a técnica utilizada foi a de avidina-biotina conjugada com peroxidase (ABC) utilizando o kit lsab-plus (DAKO, Carpinteria, EUA). O complexo foi incubado por 30 minutos à temperatura ambiente do laboratório (22°C a 25°C), e lavado com PBS da mesma maneira que anteriormente.

A revelação foi realizada com diaminobenzidina (DAB) (0,5mg/mL) e H_2_O_2_0,05% à temperatura ambiente, ao abrigo da luz. Logo após, os cortes foram lavados em água destilada, contracorados com hematoxilina de Harris e montados com Entellan.

As lâminas, nas quais foi realizada a técnica de imunohistoquímica para Gal-3, foram analisadas sob microscopia de luz comum, na objetiva de 63x, utilizando o microscópio Eclipse (Nikon, Berlim, Alemanha). A contagem de células imunomarcadas por Gal-3 foi realizada em todos os campos da musculatura intestinal. Com o auxílio de lâmina micrometrada, foi calculada a área do campo na objetiva de 63x. Em seguida, essa área (0,14µm^2^) foi multiplicada pelo número total de campos analisados para obter a área total analisada. Com o número total de células imunomarcadas e a área total analisada, foi calculada a densidade de células imunomarcadas, sendo expressa em número de células por µm^2^.

### Determinação da percentagem de colágeno

Para a avaliação da determinação da percentagem de colágeno na musculatura intestinal, foram utilizadas lâminas coradas pelo picrosírius. A coloração de picrosírius permite visualizar os colágenos tipo I e tipo III, permitindo análise qualitativa das fibras colágenas do tecido conjuntivo, por meio das diferenças de cores, intensidade e birrefringência dos tecidos corados, distinguindo principalmente as fibras tipos I e III. As fibras tipo I se apresentam grossas, altamente birrefringentes e na cor vermelha, enquanto que as fibras tipo III se apresentam em feixes finos, com fraca birrefringência e na cor amarelo-esverdeado. A análise do colágeno foi realizada em todos os campos onde foi possível observar musculatura. As imagens foram capturas utilizando um microscópico de luz comum Axio 4.1 (ZEISS, Berlim, Alemanha), uma câmera capturadora de imagem Axiocam (ZEISS, Berlim, Alemanha), um computador e o programa Axiovision 4.8 (ZEISS, Berlim, Alemanha). As imagens vistas no microscópio foram transmitidas ao monitor do computador. Para esta análise, foram utilizadas a objetiva de 40x e um filtro polarizador. Na imagem polarizada, o colágeno apresentou birrefringência com coloração amarelada, avermelhada ou esverdeada, sendo marcadas pelo examinador com auxílio de um cursor. Dessa forma, o programa determinava automaticamente a percentagem de colágeno por campo.

### Determinação da densidade de mastócitos

Para a avaliação da densidade de mastócitos, foram utilizadas lâminas coradas pela técnica de azul de toluidina. Os cortes foram desparafinados, lavados em água destilada, corados com Orange G e rapidamente imersos em álcool a 60%. Em seguida, as lâminas foram rapidamente imersas em azul de toluidina e rapidamente lavadas em água corrente. As imagens foram analisadas em um microscópico de luz comum (Nikon, Berlim, Alemanha), com objetiva de 40x.

A contagem de mastócitos foi realizada em todos os campos da lâmina. Com o auxílio de uma lâmina micrometrada, foi calculada a área de cada campo, sendo tal área multiplicada pelo número total de campos analisados, a fim de se obter a área total analisada. Com o número total de mastócitos e a área total analisada, foi calculada a densidade de mastócitos, expressa em número de mastócitos por µm^2^.

### Avaliação dos processos patológicos

A avaliação dos processos patológicos gerais foi realizada nas lâminas coradas pela coloração de hematoxilina e eosina. Foram avaliados como presente ou ausente os seguintes processo patológicos: ganglionite, miosite, congestão, hemorragia, degeneração e necrose, sendo estabelecido escore de zero e 1, onde zero corresponde a ausente e 1 a presente.

As análises histoquímica e imunohistoquímica foram realizadas por um único examinador calibrado e às cegas.

### Análise estatística

A análise estatística foi realizada por meio dos softwares *GraphPad Prism* 5 (GraphPad, San Diego, Califórnia, USA). O teste de Kolmogorov-Smirnov foi utilizado para a avaliação da normalidade. Nos casos de distribuição normal para comparação entre os três grupos, foi utilizado o teste de análise de variância (ANOVA) e, nos casos de distribuição não normal, foi empregado o teste Kruskal-Wallis. Para comparação entre chagásicos (CMI+CLM) e não chagásicos (NC) com distribuição normal, utilizou-se o teste *t* de Student e, para distribuição não normal, utilizou-se o teste de Mann-Whitney. Para comparação entre sexos, utilizou-se o teste χ^2^. Para as correlações, utilizou-se o teste de correlação de Spearman. O nível de significância assumido foi de 5% (α<0,05).

## RESULTADOS

Ao avaliar os indivíduos dos três grupos, não houve diferença significativa em relação à idade e ao sexo, demonstrando distribuição homogênea entre os grupos ( Tabela 1 ).

**Tabela 1:** Distribuição de idade e gênero dos indivíduos dos grupos chagásicos com lesão de mucosa, chagásicos com mucosa íntegra e não chagásicos

	CLM	CMI	NC
	(n=13)	(n=12)	(n=10)
Idade (Mediana) (máx-min)^*^	67 (85-44)	66 (82-54)	69 (80-56)
Gênero (M: F)^#^	4:9	4:8	6:4

^*^teste ANOVA, F=0,8458; p>0,050;^#^teste χ^2^=2350,2; p=0,309.

CLM: chagásico com lesão de mucosa; CMI: chagásico com mucosa íntegra; NC: não chagásico sem lesão de mucosa.

O Grupo CLM apresentou frequência significativamente maior de miosite (p=0,0250) e de ganglionite (p=0,0390) quando comparado aos demais. Os indivíduos chagásicos (CLM + CMI) apresentavam frequência significativamente maior de miosite (p=0,0216) e de ganglionite (p=0,0140) quando comparados ao Grupo NC ( Figura 1 e Tabela 2 ).

**Figura 1:** Ganglionite, miosite, expressão de Gal-3 e fibrose na camada muscular própria do cólon de pacientes dos grupos chagásicos com lesão de mucosa, chagásicos com muscosa íntegra e não chagásicos. A) Ganglionite no grupo chagásicos com mucosa lesada (CLM); B) Ganglionite no grupo chagásico com mucosa íntegra (CMI); C) Ausência de ganglionite no grupo não-chagásico (NC); D) Miosite no grupo chagásico com lesão de mucosa (CLM) (HE, 400x); E) Miosite no grupo chagásico com mucosa íntegra (CMI) (HE, 400x); F) Ausência de miosite no grupo chagásico com mucosa íntegra (CMI) (HE, 400x); G) Expressão de Gal-3 no grupo chagásico com lesão de mucosa (CLM) (Imunohistoquímica, 400x); H) Expressão de Gal-3 no grupo chagásico com mucosa íntegra (CMI) (Imunohistoquímica, 400x); I) Expressão de Gal-3 no grupo não chagásico (NC) (Imunohistoquímica, 400x); J) Fibrose no grupo chagásico com mucosa lesada (CLM) (Picrosírius, 1600x); K) Fibrose no grupo chagásico com mucosa íntegra (CMI) (Picrosírius, 1600x); L) Fibrose no grupo não chagásico (NC) (Picrosírius, 1600x); M) Fibrose no grupo chagásico com lesão de mucosa (CLM) (Picrosírius; imagem polarizada, 1600x); N) Fibrose no grupo chagásico com mucosa íntegra (CMI) (Picrosírius; imagem polarizada, 1600x); O) Fibrose discreta no grupo não chagásico (NC) (Picrosírius; imagem polarizada, 1600x)

**Tabela 2:** Distribuição dos processos patológicos gerais nos grupos chagásicos com mucosa lesada, chagásicos com mucosa íntegra e não chagásicos

Processos patólogicos	CLM	CMI	NC
	n=13 (100%)	n=12 (100%)	n=10 (100%)
Congestão^≠^	6 (46,15%)	10 (83,33%)	6 (60%)
Hemorragia^#^	0 (0%)	2 (16,67%)	1 (10%)
Degeneração^†^	3 (23,08%)	4 (33,33%)	2 (20%)
Necrose^‡^	0 (0%)	0 (0%)	1 (10%)
Miosite*^£^	10 (76,92%)	6 (50%)	2 (20%)
Ganglionite*^§^	6 (46,15%)	5 (41,67%)	0 (0%)

* indica diferença estatística;^≠^teste χ^2^=3.744,2; p=0,153;^#^teste exato de Fisher (CLM+CMI *versus* NC); p=1;^†^teste χ^2^=0.582; p=0,747;^‡^teste exato de Fisher (CLM+CMI *versus* NC); p=1;^£^teste χ^2^=7.347,2; p=0,025;^§^teste χ^2^=6.475,2; p=0,039.

CLM: chagásico com lesão de mucosa; CMI: chagásico com mucosa íntegra; NC: não chagásico sem lesão de mucosa.

A densidade de células imunomarcadas por anti-Gal-3 foi significativamente maior nos pacientes chagásicos (CLM + CMI) quando comparados ao Grupo NC (p=0,0032; dados não demonstrados) e significativamente maior no Grupo CMI quando comparado ao Grupo NC (p=0,0050) ( Figuras 1 e 2 ).

**Figura 2:** Densidade de células imunomarcadas por anti-galectina-3 entre os grupos chagásicos com lesão de mucosa, chagásicos com mucosa íntegra e não chagásicos * Diferença estatística de acordo com o teste Kruskal-Wallis, K=10,58; p=0,0050. CLM: chagásico com lesão de mucosa; CMI: chagásico com mucosa íntegra; NC: não chagásico sem lesão de mucosa.

Com relação à densidade de mastócitos, não houve diferença significativa entre os três grupos (p=0,5883) ( Figura 3 ).

**Figura 3:** Densidade de mastócitos entre os grupos chagásicos com lesão de mucosa, chagásicos com mucosa íntegra e não chagásicos Teste de Kruskal-Wallis, K =1,061; p=0,5883. CLM: chagásico com lesão de mucosa; CMI: chagásico com mucosa íntegra; NC: não chagásico sem lesão de mucosa.

Os pacientes chagásicos (CLM + CMI) apresentavam percentagem de colágeno significativamente maior que os pacientes do Grupo NC (p=0,0014; dados não demonstrados). A percentagem de colágeno foi significativamente maior nos Grupos CMI e CLM quando comparados ao Grupo NC (p=0,0002) com predomínio de colágeno tipo I, típico de processo inflamatório crônico, visto na cor avermelhada na luz polarizada ( Figuras 1 e 4 ).

**Figura 4:** Percentagem de colágeno entre os grupos chagásicos com lesão de mucosa, chagásicos com mucosa íntegra e não chagásicos * Diferença estatística de acordo com o teste Kruskal-Wallis, K =17,09; p=0,0002. CLM: chagásico com lesão de mucosa; CMI: chagásico com mucosa íntegra; NC: não chagásico sem lesão de mucosa.

Independentemente da integridade da mucosa, os pacientes chagásicos apresentaram maior densidade de células imunomarcadas por anti-Gal-3 e maior percentagem de colágeno quando comparados aos não chagásicos.

## DISCUSSÃO

Embora o papel biológico da Gal-3 não seja totalmente conhecido, sabe-se que ela está associada com adesão, proliferação celular e apoptose. Além disso, participa de processos patológicos, como hipertrofia celular, inflamação e transformação neoplásica.^( 22 )^Na doença de Chagas, a Gal-3 aumenta a aderência de *T. cruzi* à matriz extracelular, permitindo que os parasitas acumulem-se nesses locais antes de invadir as células hospedeiras.^( 23 )^

A Gal-3 apresenta também ação pró-inflamatória, mesmo participando da fixação de *T. cruzi* aos tecidos, estando aumentada em corações de camundongos cronicamente infectados pelo parasita.^( 12 )^Como no presente estudo, os pacientes chagásicos apresentavam densidade significativamente maior de células imunomarcadas por anti-Gal-3 quando comparados aos não chagásicos. Sugerimos que esse aumento seja em decorrência do quadro inflamatório ocasionado pelo parasita. Esse achado corrobora estudo anterior realizado pela nossa equipe, que demonstrou maior densidade de Gal-3 no megacólon dos chagásicos comparados aos não chagásicos, mas sem considerar a presença de lesões de mucosa.^( 24 )^Como os pacientes do Grupo CMI apresentaram densidade significativamente maior de Ga-3 quando comparados ao Grupo NC, reforçamos a hipótese de que essa galectina estaria aumentada devido à infecção chagásica, e não em função de possíveis lesões de mucosa, que poderiam afetar a muscular própria do cólon com consequente inflamação.

As alterações mais frequentes no megacólon chagásico são miosite, ganglionite, periganglionite, neurite, perineurite, desnervação e fenômenos degenerativos dos neurônios,^( 25 )^o que corrobora os achados do presente estudo, pois os chagásicos (CLM + CMI) possuíam presença significativamente maior de ganglionite e miosite, quando comparados aos pacientes do Grupo NC. Quando se realizou a comparação entre os três grupos, o Grupo CLM possuía presença significativamente maior de ganglionite e de miosite, quando comparado aos demais grupos. Dessa forma, o processo inflamatório nos gânglios e na musculatura intestinal do Grupo CLM deve ocorrer também pela propagação do processo inflamatório da mucosa, e não apenas pela infecção por *T. cruzi* na musculatura intestinal. Além disso, como os chagásicos apresentavam maior densidade de células imunomarcadas para Gal-3, acreditamos que essa galectina, por apresentar ação pró-inflamatória, poderia estar contribuindo para o quadro de miosite e de ganglionite. O presente estudo foi o primeiro a comparar a frequência de miosite e de ganglionite entre pacientes chagásicos com mucosa lesada e mucosa íntegra à colonoscopia.

A Gal-3 está associada também com fibrose, participando da ativação de miofibroblastos. Já foi demonstrado que a atividade fibrótica do fator de transformação do crescimento (TGF-β) só ocorre na presença de Gal-3^( 26 )^e a ausência de Gal-3 está relacionada com a interrupção do processo fibrogênico.^( 14 )^Além disso, essa galectina provoca aumento do número e degranulação de mastócitos.^( 16 )^Os mastócitos liberam triptase e trombinha, que aumentam a diferenciação de fibrócitos humanos, que, por sua vez, levam à formação de fibras colágenas em tecidos danificados.^( 17 )^No presente estudo, os pacientes chagásicos (CLM+CMI), que também apresentavam maior densidade de Gal-3, apresentavam percentagem de colágeno significativamente maior que o Grupo NC, o que corrobora com a literatura, pois já foi demonstrado em camundongos que *T. cruzi* pode ser o responsável direto ou indireto por alterações moleculares em diferentes tecidos e órgãos, podendo induzir lesões celulares, resposta inflamatória e fibrose mediada pela Gal-3.^( 27 )^No entanto, nosso estudo foi o primeiro a associar Gal-3 com fibrose em doença de Chagas em humanos.

Ao analisar individualmente os grupos, a percentagem de colágeno foi significativamente maior nos grupos CMI e CLM, quando comparados ao NC, com predomínio de colágeno tipo I, típico de processo inflamatório crônico, visto na cor avermelhada na luz polarizada. Embora não encontramos estudos que avaliaram a percentagem de colágeno comparando áreas com ou sem lesão de mucosa intestinal, sabe-se que, na doença de Chagas, existe maior percentagem de colágeno nos órgãos afetados.^( 28 )^Além disso, durante inflamação, existe liberação de substâncias que apresentam ação lítica, como metaloproteinases-2, enzimas proteolíticas que estão envolvidas na lise do colágeno,^( 29 )^o que talvez justifique o fato de o Grupo CLM ter apresentado percentagem relativamente menor de colágeno que o Grupo CMI, provavelmente em decorrência da resposta inflamatória nesses locais.

Sabe-se que, na fase crônica da doença de Chagas ocorre aumento do número de mastócitos em vários locais.^( 16 )^Estudos realizados em medula óssea de camundongos demonstraram que a Gal-3 está relacionada com o aumento do número e degranulação^( 16 )^dessas células. Portanto, levantamos a hipótese de que os chagásicos apresentassem maior densidade de mastócitos, já que apresentavam maior densidade de células imunomarcadas por Gal-3. Entretanto, no presente estudo, não houve diferença estatística em relação à densidade de mastócitos entre os grupos avaliados. Dessa forma, grande parte dos mastócitos teriam sofrido degranulação, impossibilitando que fossem identificados.

Além de estar envolvida no processo inflamatório na doença de Chagas promovendo a fixação do parasita nos tecidos, degranulação de mastócitos e fibrose, a Gal-3 está também associada com neoplasias em vários locais, estando associada com o aumento da capacidade invasiva, a diminuição da apoptose, a angiogênese e o crescimento tumoral.^( 30 )^Em cânceres colorretais, a grande expressão de Gal-3 promove o início e a progressão dos tumores, estando associada com metástase e com mau prognóstico.^( 20 )^Além disso, pacientes com aumento da expresssão de Gal-3 vão a óbito com mais frequência ou possuem maior tendência a apresentar recidivas. Por outro lado, o risco de morte é reduzido em pacientes com baixa expressão ou ausência de Gal-3.^( 21 )^Portanto, como encontramos maior densidade de Gal-3 no Grupo CMI, quando comparado ao Grupo NC, sugerimos que os pacientes chagásicos, mesmo que não apresentem lesão clínica ao exame de colonoscopia, deveriam ser acompanhados periodicamente, no sentido de prevenir o desenvolvimento de neoplasia de cólon, já que apresentam mais Gal-3 nesses locais.

## CONCLUSÃO

Pacientes chagásicos com mucosa íntegra à colonoscopia apresentaram superexpressão de Gal-3 e maior percentagem de colágeno na musculatura do cólon, quando comparados aos chagásicos com lesão de mucosa e aos não chagásicos. A maior densidade de células imunomarcadas por anti-galectina-3 nos pacientes com mucosa íntegra sugere a necessidade de acompanhamento clínico desses pacientes, uma vez que essa proteína está associada com transformação e progressão neoplásica. No entanto novos estudos devem ser realizados para compreender o mecanismo de ação da Gal-3 nos cânceres colorretais associados à doença de Chagas, a fim de sugerir as indicações e a periodicidade das colonoscopias nesses pacientes.
